# Evaluation of symphysis donor site quantity by cone-beam computed tomography and its relation to anterior loop of mental foramen: an observational study

**DOI:** 10.1186/s12903-025-06242-5

**Published:** 2025-05-28

**Authors:** Tansu Cimen, Yunus Yigit Saka, Emel Tuğba Ataman-Duruel, Onurcem Duruel, Hasan Güney Yilmaz, Tolga Fikret Tözüm

**Affiliations:** 1 Oral and Maxillofacial Radiology Faculty of Dentistry, Alanya Alaaddin Keykubat University, Antalya, 07400 Türkiye; 2 Periodontology, Faculty of Dentistry, Hacettepe University, Ankara, Türkiye; 3 Periodontology Faculty of Dentistry, Istanbul Beykent University, Istanbul, Türkiye; 4 Periodontology, Faculty of Dentistry, Near East University, Mersin, Türkiye; 5 Periodontology University of Illionois Chicago, Chicago, USA

**Keywords:** Autogenous bone grafts, Bone augmentation, CBCT imaging, Computerized tomography, Implantology

## Abstract

**Objective:**

For severely atrophic alveolar ridges, block grafting procedures are usually decided to use for reliable results. Various intraoral donor sites for autogenous block graft are presented in the literature. However, vital anatomic structures can limit intraoral bone block graft surgeries, and they must be evaluated in detail. The aim of this study is to evaluate the dimensions and volume of the maximum potential symphysis block graft donor site and their relations to the anterior loop of mental foramen.

**Materials and methods:**

According to inclusion criteria, 408 cone beam computed tomography (CBCT) images were evaluated. Mental foramen diameter, anterior loop length, the maximum potential symphysis graft dimensions (height, width, and length) and volume were measured.

**Results:**

Prevalence of anterior loop of mental foramen was calculated 30.15% (123 patients) for right and 29.66% (121 patients) for left sides. The length of anterior loop of mental foramen was measured for right and left side 4.470 ± 1.355 and 5.223 ± 1.599, respectively. The maximum potential symphysis graft height, width, length, and volume were 13.253 ± 3.261 mm, 12.694 ± 1.711 mm, 34.353 ± 2.944 mm, and 3.102 ± 1.013 cm 3, respectively. Presenting anterior loop has no significant effect on the maximum potential symphysis graft height (*P* = 0.560) and width (*P* = 0.242). However, the maximum potential symphysis block graft length (*P* = 0.017) and volume (*P* = 0.026) were decreased by increasing number of anterior loops of mental foramen.

**Conclusion:**

The mandibular symphysis bone block graft can be reliably selected as the donor site for a number of different augmentation procedures. However, there are restrictions owing to surrounding anatomical structures such as mental foramen and anterior loop. These vital structures should be considered using accurate CBCT evaluation. Presenting anterior loop of mental foramen is limiting symphysis bone block graft length and volume.

## Introduction

Dental implants are commonly used for replacing missing teeth in partial and total edentulous jaws [1, 2]. While this procedure, replacing dental implant in the most ideal position for successful prosthetic rehabilitation is sometimes limited by severely atrophic alveolar ridge in maxilla and/or mandibula [3]. In these situations, bone augmentation procedures may be required before dental implant placement for successful outcomes. Rehabilitation of osseous defects in jaws can be augmented by performing a variety of surgical procedures [4, 5]. For severely atrophic alveolar ridges, block grafting procedures are usually decided to use for reliable results [6, 7]. Numerous materials are presented for bone block grafting including, autogenous bone and bone substitutes such as xenografts, allografts, and alloplastic grafts [8–10]. For horizontal and/or vertical augmentations of extended bone defects, autogenous bone blocks are predictable [11, 12] because of its osteoinductive and osteoconductive properties with containing appropriate amount of various growth factors [13–15].

A variety of intra and extra-oral donor sites for harvesting autogenous bone blocks are available in the literature [16, 17]. The ilium, rib, calvarium, and tibia are most commonly described for extra-oral harvesting, whilst maxillary tuberosity, ramus, palatine, and symphysis are for intraoral. The advantages of using intraoral donor sites are decreasing morbidity of donor sites, decreasing number of surgical areas, being performed under local anesthesia, reducing time, and reducing cost [18, 19]. The advantage of extra-oral donor site is providing large amount of autogenous bone block graft; however, intraoral donor sites are suitable for harvest sufficient autogenous bone block while augmenting the localized alveolar ridge defects [20, 21].

Mandibular symphysis, mandibular ramus, maxillary tuberosity, and palatine are described as intraoral donor sites for oral and maxillofacial reconstruction [22]. The most preferred intraoral donor sites are the mandibular ramus and the symphysis [23, 24]. These mandibular bone blocks are used for various augmentation surgeries such as ridge augmentation, sinus augmentation, and reconstruction of alveolar clefts [19, 25]. A dense cortical bone and high amount of promoter proteins are obtained from both of these donor sites. While harvesting the symphysis graft, various instruments can be used such as fissure bur, trephines, disc, and piezoelectric devices. In addition to various instrumentations, some harvesting techniques according to patterns of the graft are presented in the literature (J-graft, ring graft, rectangular block graft, and cylindrical bone cores) [26–27]. However, vital anatomic structures such as teeth, mental and inferior alveolar nerves can limit dimensions and patterns of intraoral bone block graft surgeries, and they must be evaluated in detail.

Cone-beam computed tomography (CBCT) scan is commonly used as a diagnostic tool while planning autogenous bone block graft and dental implant procedures [28–30]. Prior to augmentation surgeries, CBCT provides the practitioners accurate knowledge about anatomic structures to avoid complications in addition to evaluating long-term healing period and osseous volume stability of bone block grafts after replacing dental implant on augmented sites [31]. Although symphysis is often preferred as intraoral donor site in oral and maxillofacial surgery, lack of study about maximum potential symphysis bone block graft volume is published in the literature [32–35]. Besides, no article evaluating the relationship between presenting anterior loop of mental foramen unilaterally or biterally and the symphysis donor site’s dimensions and volume is found in literature published in English. In the guidance of this information, the aim of this study is to present the maximum potential dimensions and volume of symphysis block that can be harvested from this region and their relations to presenting number of anterior loops.

## Materials and methods

Ethics committee approval was received for this study from Alanya Alaaddin Keykubat University Faculty of Dentistry Ethics Committee (10-2023/14-17).

### Study design

CBCT images of 408 patients, which were obtained from the patients visited the Faculty of Dentistry between January 1, 2019 and September 30, 2023, were included the study. In accordance with Helsinki Declaration 1975, as revised in 2000. All participants provided written informed consent prior to enrolment in the study. Inclusion criteria were as below: (1) no jaw fracture; (2) good visibility in the CBCT scan; and (3) no artifacts resulting from movement during image acquisition.

### CBCT image analysis

The CBCT scans were made out using KaVo OP™ 3D DVT (Kavo Dental GmbH, Biberach/Riss, Germany). Operating parameters were 90 kV and 9.23 mA, and scan time was 8.14 s with 8 × 15 cm field of view. CBCT images were evaluated by using software (OnDemand3D ^®^ version 1.0.9.1451; CyberMed, Seoul, Republic of Korea). The following parameters were noted: (1) mental foramen diameter; (2) anterior loop length; and (3) the maximum potential symphysis graft dimensions (height, width, and length) and (4) volume. All images were reviewed, and measurements performed by an experienced oral radiologist (T.Ç.).

**Fig. 1:**
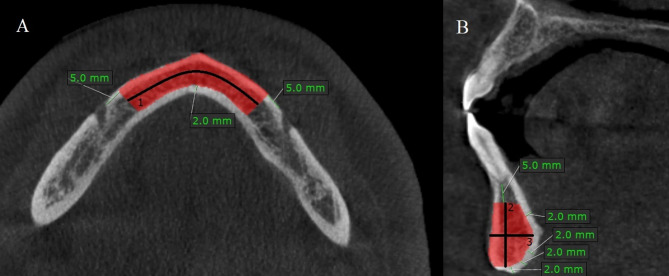
Axial (**A**) and cross-sectinal (**B**) view of maximum harvesting bone for symphysis. 1: The maximum length of the donor site was measured between 5 mm mesial to the right and left mental foramen/anterior loop. 2: The maximum height of the mandibular symphysis donor site was measured between 5 mm apical to the apex of incisor teeth and 2 mm caudal to the mandibular basis. 3: The maximum width of the donor site was measured between the buccal surface and 2 mm buccal to the lingual plate

The maximum height of the mandibular symphysis donor site was measured between 5 mm apical to the apex of incisor teeth and 2 mm caudal to the mandibular basis. The maximum width of the donor site was measured between the buccal surface and 2 mm buccal to the lingual plate. The maximum length of the donor site was measured between 5 mm mesial to the right and left mental foramen/anterior loop. Volume of maximum bone graft harvested from symphysis was calculated in cm 3 by marking the bone block in each scan. A mono-block measurement including right and left sides were made (Fig. 1).

### Statististical analysis

All statistical data were processed using a special software (IBM SPSS Statistics for Windows, Version 24.0, Armonk, NY, USA). Categorical variables were presented as the number of events and percentage. Means and standard deviation values were used for description based statistics for intermittent and continuous numeric variables. Student’s t test was used for comparing the average of independent pairs. For more than 2 independent groups, one-way ANOVAtest was utilized. Comments and assessments were based on comparisons with a statistical significance of 0.05. Inter-class correlation coefficients were performed to assess intra-observer reliability for the measurements of randomly selected 10 CBCT scans.

## Result

Intra-observer coefficients values were calculated > 90%. Four-hundred and eight CBCT images obtained from 408 patients were evaluated in the present study. Mental foramen diameter was measured for right and left side 2.072 ± 0.508 mm and 2.031 ± 0.496 mm, respectively. Prevalence of anterior loop of mental foramen was calculated 30.15% (123 patients) for right and 29.66% (121 patients) for left sides. Linear measurements of mental foramen and anterior loop of mental foramen were presented in Table 1. The maximum potential symphysis graft height, width, and length were 13.253 ± 3.261 mm, 12.694 ± 1.711 mm, and 34.353 ± 2.944 mm, respectively. The maximum potential symphysis graft volume was calculated at 3.102 ± 1.013 cm 3 (Table 1).

**Table 1:** Linear measurements and volume values of potential symphysis graft and related anatomic landmarks

Parameters	Average Values of the Measurements
Right mental foramen diameter (mm)	2.072 ± 0.508
Left mental foramen diameter (mm)	2.031 ± 0.496
Right anterior loop length (mm)	4.470 ± 1.355
Left anterior loop length (mm)	5.223 ± 1.599
Potential symphysis graft height (mm)	13.253 ± 3.261
Potential symphysis graft width (mm)	12.694 ± 1.711
Potential symphysis graft length (mm)	34.353 ± 2.944
Potential symphysis graft volume (cm^3^)	3.102 ± 1.013

Anterior loop was detected unilaterally and bilaterally in 81 patients (19.85%) and 78 patients (19.12%), respectively. No anterior loop was observed in 249 patients (61.03%). The maximum potential symphysis bone block graft dimensions and volume values according to presenting anterior loop were presented in Table 2. Presenting anterior loop has no significant effect on the maximum potential symphysis graft height (*P* = 0.560) and width (*P* = 0.242). However, the maximum potential symphysis block graft length was decreased by increasing number of anterior loops of mental foramen (*P* = 0.017). While absence of anterior loop the maximum potential symphysis block graft length was measured 34.641 ± 2.889 mm, they were 33.410 ± 2.752 mm and 32.241 ± 3.023 mm when presenting unilateral and bilateral anterior loop of mental foramen, respectively. Parallel with the maximum potential symphysis block graft length, there was a trend of decreasing the maximum potential symphysis block graft volume when increasing number of anterior loops of mental foramen (*P* = 0.026). They were 3.386 ± 1.179 mm, 3.045 ± 0.982 mm, and 2.992 ± 0.875 mm, while presenting no anterior loop, unilateral and bilateral anterior loop, respectively (Table 2).

**Table 2:** Dimensions and volume of potential symphysis graft according to presenting anterior loop

Parameters	Presence of Anterior loop			
	**No anterior loop (n = 249)**	**Unilateral (n = 81)**	**Bilateral (n = 78)**	*P* value
Potential symphysis graft height (mm)	13.123 ± 3.303	13.091 ± 3.323	13.796 ± 2.933	0.560
Potential symphysis graft width (mm)	12.668 ± 1.625	12.953 ± 1.996	12.508 ± 1.649	0.242
Potential symphysis graft length (mm)	34.641 ± 2.889	33.410 ± 2.752	32.241 ± 3.023	0.017*
Potential symphysis graft volume (cm^3^)	3.386 ± 1.179	3.045 ± 0.982	2.992 ± 0.875	0.026*

*: Statistically significance (*P* < 0.05)

## Discussion

Autogenous bone is the most preferred material for the reconstruction of osseous defects in the oral and maxillofacial regions [36]. For harvesting autogenous bone blocks, some intraoral donor sites (such as maxillary tuberosity, palatine, mandibular ramus, and mandibular symphysis) are presented in the literature as well as extra-oral donor sites [37, 38]. As a membranous bone, maxillary (tuberosity, palatine) and mandibular (symphysis, ramus) regions are excellent alternative sources for augmentation due to undergoing less resorption than endochondral bone sites. In addition, their quality and morphology can allow reconstruction of recipient site in its original contour, and harvesting of the bone graft from intraoral donor sites can be quick with minimal morbidity. When intraoral donor sites take into consideration, mandibular symphysis bone grafts have a more rapid revascularization that results in early healing, and also generally provides a larger bone volume to surgeon for harvesting in comparison with the other intraoral regions [39, 40]. Hence, the aim of this study is to present the maximum potential dimensions and volume of symphysis block that can be harvested from this region and their relations to presenting number of anterior loops.

In this study, mental foramen diameter was measured for right and left side 2.072 ± 0.508 mm and 2.031 ± 0.496 mm, respectively. According to a systematic review most recently published, the diameter of mental foramen ranged from 2.08 ± 0.53 mm to 4.44 ± 1.13 mm and it was around 3 mm most of the time [41, 42]. The findings of the present study showed similarity to the lower border of the literature range.

After symphysis bone block surgeries, the most common complication is temporary hypoesthesia and altered sensation for a particular stimulus. Prior to bone harvesting from the symphysis region, evaluation of CBCT scans can prevent potential damage to the nerve and can also aid to determine the accurate borders of the bone block. One of the crucial anatomical structures that limits our ability to harvest symphysis bone block is anterior loop. In the present study, anterior loop was detected unilaterally and bilaterally in 81 patients (19.85%) and 78 patients (19.12%), respectively. In the literature, anterior loop is detected in 38% of the subjects, of 48.4% bilaterally, of 27.8% at the right side, and of 23.8% at the left side. In addition, it is shown that there are a lot of variations about the prevalence, length, gender, and side distribution of the anterior loop in various populations [43]. Another systematic review also presents that the prevalence of anterior loop ranged from 2.47 to 94%, and the total prevalence (random effects) is 43.18% with most studies reporting bilateral localization as the most prevalent [44]. The results of the systematic review demonstrate that there is a heterogeneity between the studies on the prevalence of anterior loop and not specific for special population. However, the results of the present study are consistent with the results in the literature.

In addition to presenting anterior loop, the length of the anterior loop is also one of the crucial factor in block graft surgeries. In the present study, anterior loop lengths were found 4.470 ± 1.355 and 5.223 ± 1.599 in right and left side, respectively. The mean length of anterior loop ranged from 0.89 ± 1.17 mm to 7.61 ± 1.81 mm in the literature [41]. In another study, this value was recorded as 0.89 mm and 3.69 mm [44]. Although, there are different results in the literature, the results of the present study are compatible with the current range.

In the present study, the maximum potential height, width, and length of symphysis bone block were measured 13.253 ± 3.261 mm, 12.694 ± 1.711 mm, and 34.353 ± 2.944 mm, respectively. While Montazem et al. [34] estimated the average block sizes as 20.9 × 9.9 × 6.9 mm, Yavuz et al. [35] measured 38.75 × 11.05 × 7.8 mm. The differences between the studies can be explained by deciding on different anatomic landmarks and analyzing systems. In another study, the maximum potential height, width, and length of symphysis bone block were 13.36 ± 3.71, 8.38 ± 2.66, and 29.76 ± 7.17 mm, respectively [40]. The metholodologies of this study and the present study are the same. Minimal differences were detected between the results. However, minimal differences may be explained by different sample populations from different countries.

In the present study, the maximum potential symphysis bone block volume was calculated at 3.102 ± 1.013 cm^3^. Montazem et al. [32] calculated this volume as 4,84 mL, while Verdugo et al. [31] measured 2.3 ± 0.70 cm^3^. In another study, the volume of symphysis block graft was calculated 3.4 ± 0.9 cm^3^ [30]. Besides, Yavuz et al. [33] measured this volume 3491.08 ± 772.12 mm^3^. In another study by Ataman-Duruel et al. [40], the volume of potential symphysis graft was 3.14 ± 1.05 cm^3^. Zeltner et al. [32] assessed symphysis region by using 60 CBCT images and they presented symphysis block volume as 3.5 ± 1.3 mL. The findings of the present study show similarity to the literature.

The mandibular symphysis can be preferable intraoral graft site with its high quality and quantity. Besides, it can be frequently used as a donor site due to having no need for hospitalization and low morbidity risk. While the most suitable donor site is selected, surgeons should take into consideration some criteria as the average quantity of bone available at donor site or the quantity of the bone needed for augmentation and also restrictions due to vital structures. Prior to the augmentation surgery, planning the harvest site is essential for minimizing the risks of operation. Based on the results of this study, the mandibular symphysis bone block graft can be reliably selected as the donor site for a number of different augmentation procedures. In addition, the results of this study allow us information about the maximum potential dimensions and volume of the symphysis bone block graft along with their relation to presenting anterior loop. To the best of our knowledge, there is no study in the literature comparing the effects of anterior loop presence on the maximum potential symphysis graft dimensions and volume values. According to the results of the present study, increasing number of anterior loop has a negative effect on the potential symphysis block graft width and volume. Prior to the block graft surgeries, surgeons should evaluate CBCT scans in detail to detect limiting anatomic structures.

## Conclusion

The presence of anterior loop seems to have no significant effect on the maximum potential symphysis graft height and width, while it reduced the maximum potential symphysis graft length and volume.

## Data Availability

The datasets generated and/or analyzed during the current study are available from the corresponding author (TC) upon reasonable request.
